# Mode de révélation inhabituel d’une hyperparathyroïdie primaire du sujet jeune: à propos de deux cas

**DOI:** 10.11604/pamj.2018.30.232.12779

**Published:** 2018-07-26

**Authors:** Dhoha Ben Salah, Nabila Rekik, Lilia Affes, Mouna Elleuch, Fatma Mnif, Mouna Mnif, Mohamed Abid

**Affiliations:** 1 Service d’Endocrinologie, Diabétologie CHU Hédi Chaker Sfax, Tunisie

**Keywords:** Hyperparathyroïdie primaire juvénile, hypercalcémie, épiphysiolyse fémorale supérieure, Juvenile primary hyperparathyroidism, hypercalcemia, slipped upper femoral epiphysis

## Abstract

L'hyperparathyroïdie primaire est classique chez le sujet âgé. Les formes juvéniles sont rares et posent des problèmes diagnostiques et thérapeutiques particuliers. Nous rapportons l'observation de deux jeunes patientes, âgées respectivement de 14 et 19 ans, présentant une hyperparathyroïdie primaire révélée par une atteinte osseuse de la tête fémorale dont l'une avait une épiphysiolyse fémorale supérieure. Le retentissement était uniquement osseux dans les deux cas, sans éléments en faveur d'une forme familiale. L'hyperparathyroïdie primaire était en rapport avec un adénome parathyroïdien unique et bénin chez les deux patientes. Elles avaient bénéficié d'une adénomectomie compliquée d'une hypocalcémie persistante. Cette dernière était en rapport avec une hypoparathyroïdie aggravée par un hungry bone syndrome, nécessitant un traitement vitaminocalcique substitutif. La rareté de l'hyperparathyroïdie primaire du sujet jeune est bien classique. Le tableau clinique est dominé par les formes rénales, le mode de révélation osseux n'est pas habituel et en particulier la présence d'épiphysiolyse.

Primary hyperparathyroidism usually affects the elderly. Juvenile hyperparathyroidism is rare and poses specific diagnostic and therapeutic problems. We report the case of two young female patients, aged 14 and 19 years, with primary hyperparathyroidism detected due to femoral head involvement. One of those patients had slipped upper femoral epiphysis. Hyperparathyroidism only affected bone in both cases. No patient had a family history of this disease. Primary hyperparathyroidism was associated with solitary and benign parathyroid adenoma in both patients. They underwent complicated adenomectomy by persistent hypocalcaemia. This last was associated with hypoparathyroidism exacerbated by hungry bone syndrome, requiring substitutive vitaminocalcic therapy. It is well known that primary hyperparathyroidism rarely occurs in young subjects. Renal involvement is the most common clinical manifestation of hyperparathyroidism. Bone involvement and, in particular, slipped femoral epiphyses are an uncommon mode of revelation.

## Introduction

L'hyperparathyroïdie primaire (HPT1) est classique chez le sujet âgé. Cependant, il existe des formes juvéniles observées chez les enfants âgés de plus de huit ans, les adolescents et les adultes jeunes. Elles peuvent se présenter soit par des formes sporadiques, soit par des formes familiales isolées ou associées à une néoplasie endocrinienne multiple de type I ou de type II. Le mode de révélation habituel est le plus souvent urinaire mais aussi par la décompensation aigue d'une hypercalcémie. Par ailleurs, le mode de révélation osseux s'avère rare particulièrement avec la présence d'une épiphysiolyse de la tête fémorale. Nous rapportons le cas de deux patientes âgée respectivement de 14 et 19 ans dont le mode de révélation de l'hyperparathyroïdie était une atteinte osseuse de la tête fémorale.

## Patient et observation

**Figure 1:**
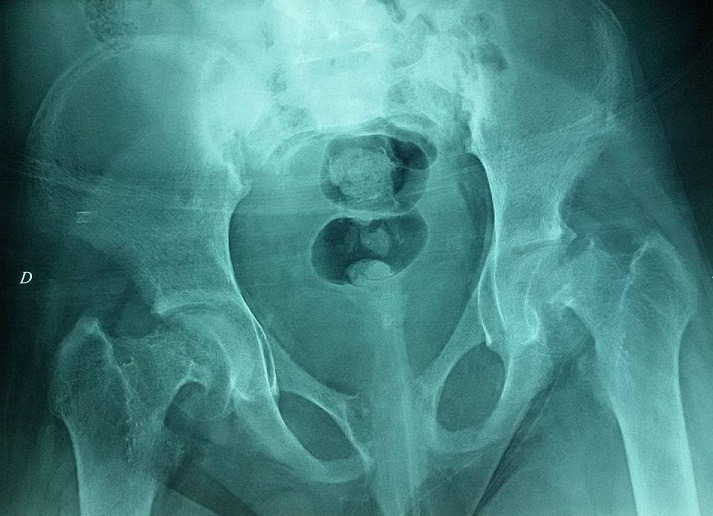
Epiphysiolyse de la tête fémorale

**Figure 2:**
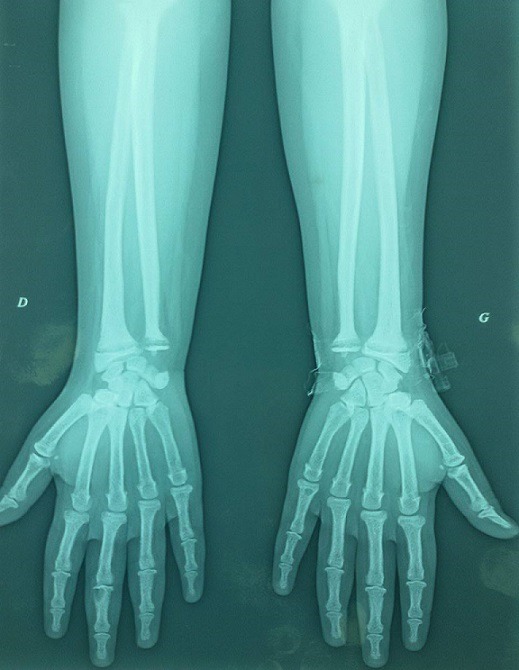
Image géodique de la main droite

**Figure 3:**
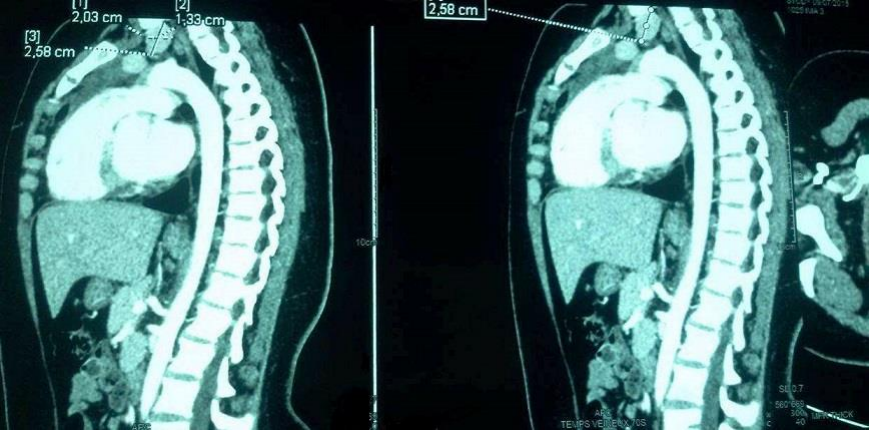
TDM cervico-thoracique: adénome parathyroïdien ectopique

**Observation 1:** Une adolescente âgée de 14 ans, sans antécédents, consulte pour douleurs au niveau de la hanche avec impotence à la marche. La radiographie du bassin avait montré une épiphysiolyse fémorale supérieur bilatérale avec une nécrose de la tête fémorale. Le bilan phosphocalcique avait montré une hypercalcémie à 3.23mmo/l, bien tolérée cliniquement. Elle était associée à une hypophosphorémie à 0.7mmol/l et une hypercalciurie à 11mmol/24h. Le dosage de la PTH a montré un taux élevé à 1496 pg/ml, associée à une hypovitaminose D < 8ng/ml. Le diagnostic d'hyperparathyroïdie primaire était alors retenu. Le retentissement était représenté par l'épiphysiolyse de la tête fémorale (EFS) (Figure 1) et la présence d'une Image géodique au niveau de la main droite (Figure 2), alors que l'osteodensitométrie était normale. L'échographie cervicale était sans anomalie. La scintigraphie montrait un nodule parathyroïdien inferieur gauche. La tomodensitométrie (TDM) thoracoabdominale montrait une masse tissulaire médiatisnale antero-superieur gauche retro et sous thyroïdienne de 2 x 2.5cm en rapport avec un adénome parathyroïdien ectopique (Figure 3). La patiente était mise sous vitamine D avec surveillance stricte de la calcémie et la calciurie. Elle était préparée en préopératoire par une diurèse forcée et deux injection de bisphosphonate a type d'acide zolédronique pour prévenir le hungry bone syndrome. Elle a bénéficiée d'une adénomectomie. L'étude anatomopathologique était en faveur d'un adénome parathyroïdien bénin. En post opératoire, elle avait présenté une hypocalcémie persistante, en rapport avec une hypoparathyroïdie aggravée par un hungry bone syndrome. Un traitement vitaminocalcique substitutif était alors prescrit. L'épiphysiolyse a été respectée et l'évolution était favorable.

**Figure 4:**
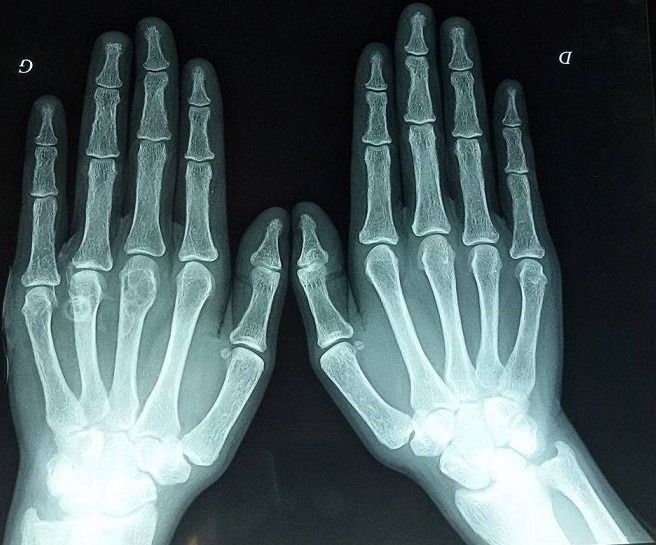
Tumeur brune de la main gauche

**Observation 2:** Une jeune fille âgée de 19 ans, sans antécédents, consulte pour douleurs et limitation à la mobilisation des hanches, associées à une asthénie et un amaigrissement depuis un an. L'examen clinique montrait une altération de l'état général avec quelques signes de déshydratation extracellulaire et des douleurs à la mobilisation active et passive de la hanche. A la biologie elle présentait une hypercalcémie à 3.13mmo/l et une hypophosphorémie à 0.6mmol/l associée à une hypercalciurie à 14mmol/24h. La PTH était élevée à 1800 pg/ml, associée à une hypovitaminose D à 5ng/ml. Le diagnostic d'HPT1 était retenu. La patiente était mise sous vitamine D et diurèse forcée en surveillant la calcémie, la calciurie et l'état d'hydratation. Le retentissement était aussi uniquement osseux avec la présence de fractures pathologiques des deux cols de fémur non déplacées et une tumeur brune au niveau de la main gauche (Figure 4). L'osteodensitométrie montrait une ostéoporose avec un Z Score à -6,4. L'échographie cervicale montrait une masse parathyroïdienne droite de 3cm fixante à la scintigraphie. La patiente a bénéficié d'une adénomectomie après normalisation de la calcémie et prise de deux injections de bisphosphonate. L'étude anatomopathologique était en faveur d'un adénome parathyroïdien bénin. Les suites opératoires étaient comparables à la première patiente. La conduite pour les fractures était le repos et la marche avec béquilles. L'évolution à 3 mois postopératoire était favorable avec consolidation des fractures. La recherche d'une forme familiale chez les deux patientes était négative.

## Discussion

L'HPT 1 du sujet jeune est une entité classiquement rare. Son individualité a été bien mise en valeur par plusieurs études récentes dans les littératures [1-7]. Elle est à différencié des exceptionnelles HPT 1 néonatales ou du très jeune enfant secondaire à une hyperplasie glandulaire diffuse [8]. Etant donné la fréquence des formes asymptomatiques, la non spécificité des symptômes dans la majorité des cas et l`évolution lente de la maladie, le diagnostic et la prise en charge des patients atteint d'HPT1 sont généralement retardés. A l'interrogatoire, il est habituel de retrouver des symptômes évoluant depuis plusieurs années ou la notion de lithiases rénales récidivantes anciennes. Il est donc probable que l'HPT1 juvénile est sous-évaluée car méconnue chez l'adolescent et l'adulte jeune [9]. La symptomatologie clinique est caractérisée par la fréquence des formes rénales associant néphrocalcinose et lithiases urinaires récidivantes. Ces dernières sont retrouvées dans les deux tiers des cas dans la série de O.Monneuse [10] et dans 40% des cas dans la série de la Mayo Clinic [3]. Ce qui différencie les HPT 1 juvéniles des HPT 1 du sujet âgé qui sont le plus souvent traitées à un stade asymptomatique ou révélées par des symptômes osseux prédominants. Le mode de révélation osseux ou ostéomusculaire est rare chez l'enfant dans la littérature, avec une prévalence de 4% dans la série de O. Monneuse [10, 11]. Comme ce fut le cas de nos deux patientes. Il faut aussi insister sur la fréquence du mode de révélation aigue chez ces sujets jeunes en rapport avec une hypercalcémie aigue qui traduit l'agressivité de la maladie. Elle survient chez 50% des enfants âgés de moins de 12 ans [1], et dans 12% des cas dans la série de O. Monneuse [10]. Concernant les formes osseuses rares, l'association entre HPT1 et EFS est exceptionnellement décrite dans la littérature [12, 13]. L'HPT1 est une cause rare d'EFS, dont seulement 11 cas ont été signalés auparavant jusque-là [12, 13]. L'effet osseux de la PTH est bien connu, mais la découverte de récepteurs à la PTH au niveau de la zone de croissance des chondrocytes est une nouvelle entité [14]. Les récepteurs à la PTH sont présents en grand nombre dans les cellules de la zone de cartilage épiphysaire hypertrophié, c'est à ce niveau où survient l'épiphysiolyse [14].

La PTH joue un rôle important dans la stimulation de diverses métalloprotéinases responsables de l'ossification du cartilage, et contrôlent la dégradation de la matrice du cartilage pendant la formation osseuse [15]. Ces actions sur les chondrocytes de la plaque de croissance, par l'intermédiaire de ses médiateurs et de leurs métalloprotéinases, peuvent être perturbées par tout déséquilibre de la PTH, ce qui entraîne une minéralisation anormale du cartilage et allonge le temps nécessaire à l'achèvement de la fusion épiphysaire [16]. Cette période allongée pendant laquelle le cartilage reste non calcifié peut être l'un des facteurs favorisant le développement de l'EFS. L'élévation du taux de PTH peut déclencher donc le processus de l'épiphysiolyse notamment en présence d'autres facteurs déstabilisants la plaque de croissance (Activité physique intense, obésité, traumatisme, etc.) pendant une longue période [16]. Le déficit en vitamine D est fréquemment observé chez les patients atteints d'HPT1. Il aggrave dans ce contexte l'EFS, qui a son tour peut être secondaire au déficit en vit D isolé [17]. Chez notre patiente le déficit en vit D est un facteur aggravant le retentissement osseux de la PTH et l'épiphysiolyse. Cette association impose un traitement soigneux de l'hypovitaminose D [18]. La prise en charge des EFS instable doit être prudente car il existe un risque important de développer une nécrose avasculaire de l'articulation. Cependant, le moment optimal pour réparer l'articulation reste controversé [18]. Les manifestations cliniques et les complications de l'hypercalcémie conditionnent le traitement. En cas d'anomalies cardiaques, de symptômes neurologiques ou d'atteintes musculo-squelettiques, une parathyroidectomie devrait être prioritaire à la fixation de l'EFS [12]. Dans notre cas, la parathyroidectomie a été effectuée avec respect de l'épiphysiolyse. En effet, la parathyroidectomie peut permettre, à elle seule, la guérison de l'épiphysiolyse [19]. En 2012, El Scheich et al ont rapporté le cas d'un patient avec une HPT1 associé à une EFS [12]. Ils ont conçu une approche algorithmique de prise en charge pour ces patients [12]. Cette dernière et d'un apport considérable pour les cliniciens et les chirurgiens. Enfin le diagnostic doit faire rechercher de principe une néoplasie endocrinienne multiple, principalement de type 1. En effet le pourcentage des NEM 1 dans tout HPT1 est de 1 à 18% [20]. Cette fréquence est inversement proportionnelle avec l'âge avec un maximum avant 30 ans (20) d'où un dépistage systématique dans cette tranche d'âge. Cependant, dans les formes juvéniles l'association a une NEM1 est exceptionnelle. Dans la série de O.Monneuse [10], l'HPT 1 n'a jamais révélé la NEM, comme le cas de nos deux patientes.

## Conclusion

Dans l'HPT 1 du sujet jeune, Le tableau clinique est dominé de façon très caractéristique par les formes rénales. Le mode de révélation osseux n'est pas habituel et en particulier la présence d'épiphysiolyse. Cette association rare et ses manifestations devraient être gérées efficacement en fonction du profil clinique du patient pour améliorer la qualité de vie et le pronostic de ces patients. Dans ce cadre une stratégie thérapeutique bien codifiée s'avère indispensable.

## Conflits d’intérêts

Les auteurs ne déclarent aucun conflit d'intérêts.
